# An Assessment on Average Pressure Drop and Dust-Holding Capacity of Hollow-Fiber Membranes in Air Filtration

**DOI:** 10.3390/membranes11070467

**Published:** 2021-06-24

**Authors:** Pavel Bulejko, Ondřej Krištof, Miroslav Dohnal

**Affiliations:** 1 Heat Transfer and Fluid Flow Laboratory, Faculty of Mechanical Engineering, Brno University of Technology, 61669 Brno, Czech Republic; 2 ZENA Membranes s.r.o., 62100 Brno, Czech Republic; ondrej.kristof@vut.cz (O.K.); dohnalml@zena-membranes.cz (M.D.)

**Keywords:** hollow-fiber membrane, air filtration, pressure drop, dust-holding capacity

## Abstract

In this work, we tried to analyze dust loading behavior of polypropylene hollow fiber membranes using average pressure drop models. Hollow fiber membranes varying in fiber diameter were loaded with a standardized test dust to simulate particle-polluted air. We measured pressure drop development of the membranes at different flowrates and dust concentrations, and, after each experiment, the dust deposited on the membrane fibers was weighed to obtain dust holding capacity (DHC). The obtained experimental data was analyzed using various average pressure drop models and compared with average pressure drop obtained from pressure drop/dust load dependence using a curve fit. Exponential and polynomial fitting was used and compared. Pressure drop in relation to the dust load followed different trends depending on the experimental conditions and inner fiber diameter. At higher flowrate, the dependence was polynomial no matter what the fiber diameter. However, with higher fiber diameter at lower permeate velocities, the dependence was close to exponential curve and followed similar trends as observed in planar filter media. Dust-holding capacity of the membranes depended on the experimental conditions and was up to 21.4 g. However, higher dust holding capacity was impossible to reach no matter the experiment duration due to self-cleaning ability of the tested membranes.

## 1. Introduction

Air filters and membranes have been applied to many chemical engineering processes to provide off-gas treatment and high purity gases/compressed air. They are further ubiquitous in HVAC (heating, ventilation and air conditioning) applications especially for health risk reduction associated with polluted air in buildings [1] and protection of related equipment [2]. To choose a correct filter for different applications, it is necessary to know several characteristics such as filter area, filtration efficiency, capacity to capture dust particles, and pressure loss. The latter is critical as it determines the energy use which accounts for about 75% of total air filtration cost.

The development of pressure loss over a filter media with particle fouling is an essential issue in practically all filtration applications. As particles deposit inside the filter or onto the filter surface, the filter resistance increases, thus increasing the necessary performance of a fan, pump, or other equipment used to draw the fluid. A repercussion of this can be observed in increasing power demand to operate such equipment, hence the operation cost, which is essential when designing filtration systems [3]. Pressure drop evolution during particle loading and an ability to keep the deposited dust are important characteristics of every filter which determines the requirements on accessories used in air filtration. Therefore, a study of such characteristics is mostly included in standardized test methods for air filters and aims to obtain information about average pressure drop over lifetime of the filter and ability to capture dust. This is mostly done using an accelerated particle loading which aims to simulate real conditions. In reality, when the pressure drop is at a certain level, mostly double the initial value, the filter is changed for a new one. Membrane filters can be repeatedly cleaned by back-pulse, back-blow [4,5], or simply by air-blowing the surface. This is an advantage as membrane filters have generally higher initial pressure drop. Such a cleaning is also possible in hollow-fiber membranes.

Hollow-fiber membranes are of increasing importance in the air filtration field, even though they have been studied very little so far. Most of the studies about this issue often aimed at hollow-fiber membranes in terms of materials science and engineering, i.e., their manufacturing from different materials using various preparation techniques and characterization on filtration efficiency and pressure drop. Some works tried to do some mathematical modelling related to this issue [6,7]. However, only two works fully focused on pressure drop evolution during particle loading at different experimental conditions [8,9]. The work by Wang et al. [8] focused on the effects of particle hygroscopicity and relative humidity (RH) on particle loading behavior of hydrophilic/hydrophobic (PAN/PVDF) hollow fiber membranes. They observed that, when RH was high, hydrophobic filters had a dramatic pressure drop increment at a later stage while hydrophilic filters had a quick rise in the pressure drop at the initial stage. When RH was low, the filter pressure drop was independent of particle hygroscopicity and membrane hydrophilicity. In another work [9], the authors focused on loading behavior of polypropylene hollow-fiber membranes at different experimental conditions. They further compared the available mathematical models for cake pressure drop with the experimental results. They found that membrane fouling is very slow and the membranes have self-cleaning ability. Moreover, the basic prediction models for cake pressure drop such as Carman–Kozeny or Ergun equation were in a strong disagreement with the experimental data. As this issue is quite crucial in the use of air filters, further works are necessary.

The main motivation of the study is to get a general idea about dust loading behavior and dust-holding capacity of hollow-fiber membranes, which has not been done so far. Furthermore, it is necessary to compare available average pressure drop models with the experimental data. This is generally known for planar filter media, for which the models were used and verified. However, despite the increasing amount of publications on the use of hollow-fiber membranes in air filtration, the particle loading behavior and especially dust-holding capacity, was practically not studied. Only two works were found to mention and measure dust-holding capacity [10,11]. However, this problem was addressed rather marginally. Therefore, we tested hollow-fiber membranes with different geometrical parameters in filtration experiments with standardized ASHRAE A2 fine test dust at a constant air flowrate. We measured pressure drop during continuous dust loading until reaching double the initial pressure drop. After each experiment, the mass of deposited dust was measured to obtain dust-holding capacity (DHC). The obtained data were analyzed using average pressure drop models and compared among each other, among different membranes, dust loading rates, and airflow velocities.

## 2. Analysis on Average Pressure Drop of Air Filters

During a real air filtration process, pressure drop development with dust particle deposition varies depending on parameters of filter, dust (amount), flowrate, and air properties according to the general equation:

(1) $$\Delta p_{\mathrm{avg}}=f\left(Q,K,T,H,m\left(t\right)\right)$$

where Δ*p*_avg_ is the average pressure drop and *Q*, *T*, *H*, *m*(*t*), and *K*, respectively, are the air flowrate, air temperature, air humidity, and dust amount loaded at a given time and a constant describing dust and/or filter type. The overall process is quite complex and is often simplified using various averaging models including the arithmetic model, which is a simple arithmetic average of initial (pressure drop of a new filter, Δ*p*_i_) and final pressure drop (Δ*p*_f_) as follows:

(2) $$\Delta p_{\mathrm{avg}}=\frac{\Delta p_{i}+\Delta p_{f}}{2}$$

then a geometric model, which is the geometric mean of the initial and final pressure drop:

(3) $$\Delta p_{\mathrm{avg}}=\sqrt{\Delta p_{i}\Delta p_{f}}$$

and an integral model:

(4) $$\Delta p_{\mathrm{avg}}=\Delta p_{i}+\frac{\Delta p_{f}-\Delta p_{i}}{3}$$

The three models above are considered a weighted average of pressure drop curve using initial and final pressure drop. Such a simplification, however, could bring an error into the calculations. Hence, a dust loading test must be performed to obtain more accurate results and a curve fitting must be used. Exponential curve fit for dependence of pressure drop on dust load was found through testing various filters and is of a common form [12]:

(5) $$\Delta p\left(m\right)=ae^{bm}$$

where Δ*p*(*m*) is the instantaneous pressure drop across the filter as a function of dust load, *a* and *b* are the regression parameters, and *m* is the instantaneous mass of dust deposited:

(6) $$m=Qt\eta C_{\mathrm{up}}$$

where *t*, *η*, and *C*_up_ are instant time of filtration, filtration efficiency, and upstream particle concentration, respectively. Equation (6) can be used to estimate instant dust mass deposited on the surface of planar filter media. The parameter *a* expresses the distance of the loading curve intersection on the *y*-axis from zero, which can be approximated by initial pressure drop Δ*p*_i_:

(7) $$a=\Delta p_{i}$$

while *b* can be expressed from logarithmized Equation (5) and setting required conditions. The average pressure drop can also be modeled by a 4th order polynomial according to 4/11 Eurovent calculation [13] as previously used by several researchers [14,15]:

(8) $$\Delta p\left(m\right)=vm^{4}+xm^{3}+ym^{2}+zm+\Delta p_{i}$$

where *v*, *x*, *y*, and *z* are the fit parameters of the polynomial. Average pressure drop is a function of dust load and can be generally described as follows:

(9) $$\Delta p_{\mathrm{avg}}=\frac{1}{M}\int_{0}^{M}\Delta p\left(m\right)dm$$

where *M* is the final amount of dust loaded. The equation for the average pressure drop is then obtained by integrating the equation for instantaneous pressure drop (Equation (5) or (8)) to the final dust load [12,16]:

(10) $$\Delta p_{\mathrm{avg}}=\frac{1}{M}\int_{0}^{M}\Delta p_{i}e^{bm}dm=\frac{\Delta p_{i}}{bM}\left(e^{bM}-1\right)$$

when using exponential curve fit (Equation (5)) or:

(11) $$\begin{array}{ll} \Delta p_{\mathrm{avg}} & =\frac{1}{M}\int_{0}^{M}\left(vm^{4}+xm^{3}+ym^{2}+zm+\Delta p_{i}\right)dm\\ & =\frac{1}{5}vM^{4}+\frac{1}{4}xM^{3}+\frac{1}{3}yM^{2}+\frac{1}{2}zM+\Delta p_{i} \end{array}$$

when using a polynomial fitting, i.e., Equation (8). The above relationships do not indicate anything about how long the filter should operate until exchange for a new filter and neglects the rate of particle accumulation in the filter. An estimate of pressure drop at change-out of the filter can be expressed as follows [17]:

(12) $$\Delta p_{c-o}=\Delta p_{i}\exp\left[\frac{Qt_{f}\eta C_{\mathrm{up}}}{DHC}\ln\left(\frac{\Delta p_{f}}{\Delta p_{i}}\right)\right]$$

where *DHC* is the filter dust holding capacity at the final pressure drop Δ*p*_f_.

## 3. Materials and Methods

### 3.1. Hollow-Fiber Membrane Bundles

**Figure 1:**
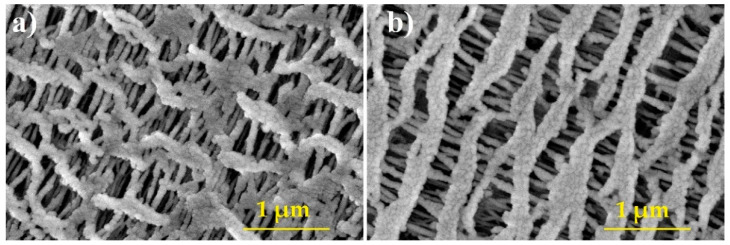
Pore structure of tested hollow-fiber membranes P60 (**a**) P80 (**b**).

Hollow-fiber membranes of different fiber diameter and surface area (Table 1) provided by Zena Membranes s.r.o. [18] were tested (Figure 1). These are made of polypropylene by extrusion and subsequent dry stretching technique. Such production is relatively simple, with no waste material produced. The polypropylene hollow-fiber membranes are naturally hydrophobic and have been used in various mainly liquid treatment applications including water/wastewater treatment, membrane bioreactors/contactors [19], membrane distillation [20], and medicine/biochemical laboratory uses.

### 3.2. Experimental Setup

**Figure 2:**
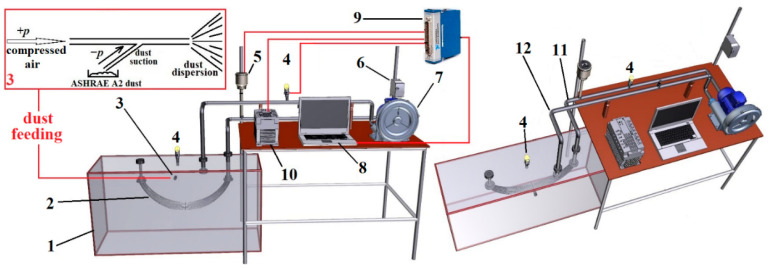
A scheme of the experimental setup: **1**—chamber, **2**—hollow-fiber membrane bundle, **3**—opening for dust dosing, **4**—differential pressure sensor, **5**—air velocity probe, **6**—inverter, **7**—blower, **8**—laptop, **9**—DAQ card, **10**—laboratory power source, **11**—air intake (downstream) pipe, **12**—air return (exhaust) pipe.

A scheme of the experimental setup is shown in (Figure 2). Filtration experiments were done in a transparent chamber (1) connected to a fan (7) using a suction pipe (11), to which a hollow-fiber membrane bundle (2) was linked. Dust feeding was done via an opening (3) in the wall of the chamber using a pressure air nozzle (3 in red frame in Figure 2) as described elsewhere [10]. The suction pipe (11) was provided with an EE660 air velocity probe (E+E Elektronik Ges.m.b.H., Engerwitzdorf, Austria) (5) and an Omega PX277-05D5V differential pressure transducer (OMEGA Engineering Inc., Norwalk, CT, USA) (4) to measure pressure drop of the hollow-fiber membrane during dust loading. The fan frequency was controlled using a Sinamics V20 inverter (Siemens AG, Munich, Germany) (6). LabView software was used for data acquisition from the sensors via a 9207 C Series Voltage/Current input module (National Instruments Corp., Austin, TX, USA) (9) connected to a laptop (8). Sensors and DAQ module were powered using a HY3003D laboratory DC power supply (Shenzhen Huayi Peakmeter Technology Co., Ltd., Shenzhen, China) (10). Data were recorded with a frequency of 0.5 Hz (one point per 2 s).

### 3.3. Dust Supply Scheme

**Figure 3:**
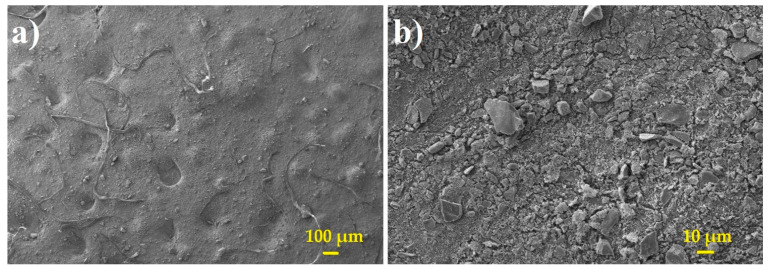
SEM image of ASHRAE A2 dust used in filtration experiments at magnification 100× (**a**) and 1000× (**b**).

The dust was fed into a closed chamber via an orifice, and we tried to create a sufficient dusty atmosphere to expose the membrane bundle during the filtration experiments. It was necessary to choose a suitable dust feeding rate (amount of dust fed per unit time) to keep an abundant amount of dust airborne for a sufficiently long period of time so we can obtain at least roughly defined dust concentration (amount of dust per unit volume of air). The dosing scheme was based on an estimate of sedimentation velocity of ASHRAE A2 dust particles (Figure 3), which were fed into the experimental chamber. However, it was necessary to keep constant dust concentration for a certain time until the next dust dose. Hence, we had to estimate how long the particles were kept airborne prior to the sedimentation and available to the membrane. This means to make an estimate of sedimentation velocity based on the size and density of the ASHRAE dust particles.

In this study, a value of geometric mean diameter as large as 2.62 µm and a particle density of 2100 kg/m^3^ [9] were used to calculate the particle sedimentation velocity *v*_p_ as follows:

(13) $$\nu_{p}=\sqrt{\frac{4}{3}\frac{\left(\rho_{p}-\rho \right)gd_{p}}{\xi \rho }}$$

where *d*_c_, *ρ*_c_, *ρ*, *g*, and *ξ* are the particle diameter, particle density, fluid density, gravitational acceleration, and friction coefficient. Friction coefficient depends on the sedimentation profile which can be characterized by Reynolds number *Re* = *d*_p_*v*_p_*ρ*/*µ*. However, we can see that the sedimentation velocity we need to estimate is included in the Reynolds number formula. Therefore, the Archimedes number must be introduced to assess the character of sedimentation:

(14) $$Ar=\frac{d_{p}^{3}\left(\rho_{p}-\rho \right)\rho g}{\mu^{2}}$$

where *µ* is the fluid viscosity. The Archimedes number was lower than 3.6 indicating laminar character of sedimentation; the Reynolds number is then *Re* = *Ar*/18, so the following formula can be used to calculate the friction coefficient:

(15) $$\xi =\frac{24}{Re}=\frac{432}{Ar}$$

The sedimentation velocity was as high as 0.43 mm/s. Based on this value and the height of the chamber (400 mm), a dosing interval of 15 min was chosen to be enough for particles to be continuously airborne until the next dust dose. Therefore, during the filtration experiment, the membrane bundle was continuously surrounded by particles in a sufficient concentration prior to their sedimentation to the bottom of the chamber. Hence, we could consider the particle concentration constant during the 15 min interval and thus compare among different dust loading rates.

## 4. Results and Discussion

### 4.1. Experimental Data

Table 2 shows an overview of the obtained experimental data, i.e., experimental configuration (conditions), measured dust-holding capacity (*DHC*_meas_), initial (Δ*p*_i_) and final pressure drop (Δ*p*_f_), the total amount of dust fed during the experiments (*M*), and experiment duration (*t*). We did experiments at a constant dust loading rate of 4 g/h with two different membranes (refer to Table 1) at different permeate velocity (a velocity of the air stream within the pipe at the downstream side of the membrane, refer to Figure 2, part 11—air intake/suction pipe). Based on the series of these experiments, we further chose to assess the P80 membrane at a constant permeate velocity of 20 cm/s but at three different dust loading rates (2, 4 and 6 g/h). This was done to compare the influence of dust concentration on the loading behavior of the P80 membrane in terms of average pressure drop calculated using different models. P80 at 20 cm/s was chosen to further evaluate due to an optimal loading behavior similar to the pressure drop/dust load trends observed in planar filters. The data obtained for this membrane was thus possible to approximate using the exponential curve fit, which is generally done in the ASHRAE dust-holding capacity test [21] and which was previously used in several studies [12,17]. We could then better compare among different average pressure drop models including the exponential, even though, for the other experimental configurations, the exponential curve did not fit well and polynomial dependence must have been used instead. However, both of these curve fits were used to approximate obtained data (no matter of fitting accuracy) and compared for each experiment configuration to better see the variations in the results obtained using different average pressure drop models.

In planar filters, practically all dust carried by an air stream is deposited on the surface, except the particles, which penetrate through the filter. Thus, the real dust amount captured and kept on the filter surface is related to the filter efficiency (dust arrestance) according to Equation (6). Unlike in planar filters, only a partial amount of dust is deposited on the HFM surface while the remaining dispersed dust surrounding the membrane bundle settles down in the chamber [22]. Therefore, it was necessary to estimate the rate of dust deposition on the HFM bundle at given conditions. However, the hollow fiber geometry complicates this task as the deposited dust will differ in every instant during the experiments as the membrane surface area decreases due to membrane fouling by dust particles. As an approximation, we used the experimental DHC results (Table 2) to estimate the amount of dust deposited in time causing the given pressure drop increase as follows:

(16) $$m_{\mathrm{HFM}}=\frac{DHC_{\mathrm{meas}}}{A}\frac{t}{t_{f}}$$

where *DHC*_meas_ is the experimentally obtained dust-holding capacity, *A* is the membrane surface area, and *t*_f_ is the final time of filtration.

**Figure 4:**
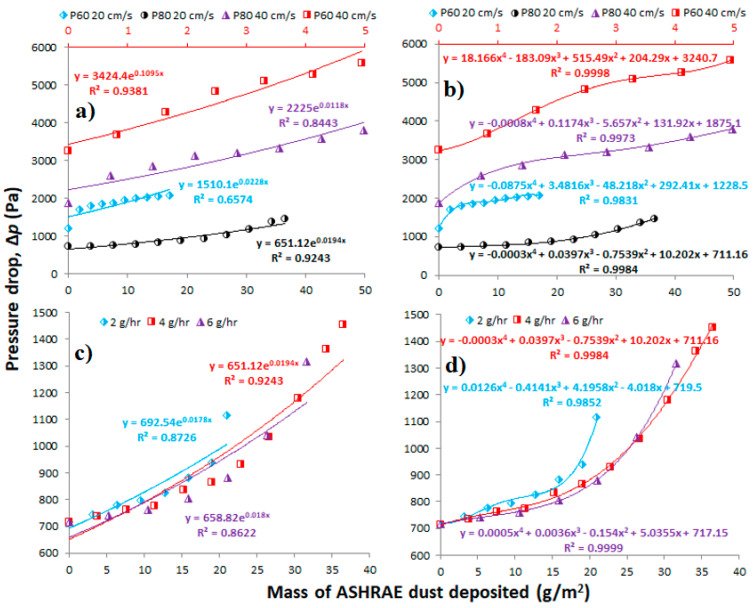
Dust loading curves fitted with different models: (**a**) various membranes and velocities at a constant loading rate of 4 g/h with exponential fit (the minor *x*-axis in red is for P60, at 40 cm/s), (**b**) the same fitted with 4th order polynomial (the minor *x*-axis in red is for P60, at 40 cm/s), (**c**) P80 membrane at 20 cm/s at different loading rates with exponential fit, (**d**) the same fitted with 4th order polynomial.

Figure 4 shows the dust loading curves approximated using exponential (Figure 4a,c) and polynomial (Figure 4b,d) curve fits. Compared to the exponential interpolation (Equation (10)), we can see that the polynomial fitting (Equation (11)) better approximates the data from all experiments. The exponential curve fits the best to the data of P80 membrane at 20 cm/s. The larger fiber diameter (0.6 mm) of the hollow fibers in this membrane causes significantly lower pressure drop compared to P60 membrane with a fiber diameter as large as 0.3 mm, which is in accordance with the Hagen–Poiseuille equation [9]. Hence, the dust loading behavior for this membrane is similar to planar or pleated filter media which generally have very low initial pressure drops as observed in other studies [12,17]. The exponential curve fit (Equation (10)) is mostly used in the ASHRAE 52.2 method [21] to simulate real pressure drop curve in relation to dust load [16] and is better used for filters used in general ventilation. Polynomial approximation thus appears to be more universal as it can be used to fit various loading curves more accurately. This is also obvious throughout the literature where polynomial fit is reliably used to optimize and analyze performance of filters in various applications such as airliner cabin filters [23], engine air intake [15], or evaluating the HEPA filter media [14]. Comparing the coefficients of determination (R^2^), we can see better tightness of polynomial fit compared to the exponential. We can also compare the absolute terms, which in both models correspond to the values of membrane initial pressure drop. The absolute terms from both models are different by more than 200 Pa in some cases, so we can assume that different fitting after integrating the equation will provide quite different values of dust mass-averaged pressure drop.

### 4.2. Average Pressure Drop

**Figure 5:**
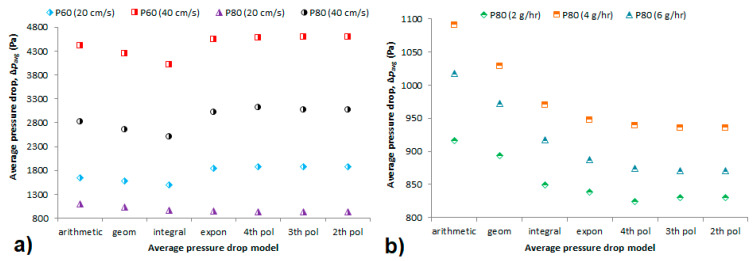
Average pressure drop of different membranes at different permeate velocities at a constant loading rate of 4 g/h (**a**) and P80 membrane at 20 cm/s at different loading rates (**b**).

Figure 5 shows the average pressure drop obtained using different models and predicted pressure drop at which the filter should be changed out (Δ*p_c-o_*). The first three average pressure drops (arithmetic, geometric and integral) are calculated solely based on experimental values of initial pressure drop of the membrane (clean membrane at the beginning of the experiment) and final pressure drop after dust loading (membrane heavily loaded with dust at the end of the experiments). They do not include the influence of gradual dust accumulation in the filter, which, according to Montgomery et al. [17], can bring an error in the average pressure drop results and thus underestimate the power demand and supply when designing a filtration system [24]. In different experiments at a loading rate of 4 g/h (refer to Figure 5a), we can see the average pressure drop values decrease in order (model) polynomial–exponential–arithmetic–geometric–integral in all experiments, except P80 membrane at 20 cm/s. Excluding the P80 at 20 cm/s, this is quite different from previous work [12] aiming at a comparison of different filter media, namely a V-pack filter, box filter, bag filter, and panel filter. All these filter media had average pressure drop based on the exponential model comparable to that calculated using the integral model or slightly higher. Thus, the integral model was considered reliable to predict the average pressure drop of the tested filters.

The exponential model (Equation (10)) can be used as a correction of the arithmetic average pressure drop including the effect of gradual dust accumulation on the hollow-fiber membrane. The average pressure drop after correction using the exponential model is up to 12% higher compared to arithmetic average. This is in accordance with literature [17] as this model was used to predict pressure drop of planar filter media with an accuracy of ±10%. The average pressure drop based on the polynomial model is even higher than the results obtained using the exponential fit, again except P80 membrane at 20 cm/s, also for different loading rates. However, these results could be considered the most accurate due to tight curve fit to the experimental data.

Concerning the P80 at 20 cm/s for different loading rates (2, 4, 6 g/h, Figure 5b), the average pressure drop decreases in arithmetic–geometric–integral–exponential–polynomial order, which is similar to that observed in fibrous (planar, pleated) filter media [12]. This could be explained mainly by the hollow fiber geometry, which causes additional pressure drop due to fluid friction along the narrow lumen side of the hollow fiber. This means that pressure drop along the fiber length dominates over the pressure drop caused by deposited dust on the membrane surface. This effect is less apparent in the P80 membrane, which has larger fiber diameter and at lower permeate velocity (it is not valid for higher velocity for which the pressure drop along the fiber lumen also dominates over the pressure drop caused by dust cake). Hence, the values of pressure drop based on polynomial and exponential model are lower for this membrane as the effect of the pressure drop over the length of the fiber is not accounted for in the parameters of the equations of respective fitting models. In the other experiments, it can be observed vice versa. Based on the above-mentioned, we can see that the average pressure drops calculated using different models may vary significantly. Erroneous calculation of average pressure drop can mislead the balancing of the power demand and supply when designing a filtration system, which increases the operation costs quite abruptly [24].

### 4.3. Dust-Holding Capacity

Dust-holding capacity (DHC) is the amount of dust kept on the filter after dust loading at the final pressure drop [25]. Final pressure drop is mostly considered as twice the initial pressure drop value in high efficiency filter media [26]. Here, we measured the weight of dust deposited on the hollow-fiber membrane bundle after each experiment at double the initial pressure drop value, if allowed by the experimental conditions (for example, the experiment with P60 membrane at 40 cm/s was intermitted prior to reaching the double the initial pressure drop, i.e., after 6 h, due to the blower overheating). DHC is dependent on many parameters including filter area and efficiency, filtration velocity (flowrate), dust concentration, and duration of the filter use. Based on the above-mentioned parameters, we can estimate DHC theoretically using the same formula as Equation (6) but with time at the end of filtration experiment (*t_f_*) or more generally time when the filter reaches the final pressure drop Δ*p*_f_ and related to unit membrane area (A):

(17) $$DHC_{\mathrm{theor}}=\frac{Qt_{f}\eta C_{\mathrm{up}}}{A}$$

However, this relationship is usable for planar or pleated filters only, as a vast majority of dust is deposited on the filter surface (except dust, which penetrates through the filter depending on the filter efficiency). In hollow-fiber geometry, only a partial amount of the dosed dust is deposited on the fibers surface. More accurate values may be obtained with the experimental data fitted with exponential curve using the exponential regression parameter *b*, which can be derived from logarithmized Equation (5) as follows:

(18) $$b=\frac{1}{m}\ln\frac{\Delta p\left(m\right)}{a}$$

Inserting the Equation (7) into Equation (18) and putting the following conditions

*a* = Δ*p*_i_ (19)

*m* = *DHC* (20)

we therefore obtain:

Δ*p*(*m*) = Δ*p*(*DHC*) → Δ*p*_f_ (21)

which is pressure drop at dust-holding capacity, i.e., final pressure drop, we get:

(22) $$b=\frac{1}{DHC}\ln\frac{\Delta p_{f}}{\Delta p_{i}}$$

Rearranging Equation (22), we can obtain dust-holding capacity based on exponential regression as follows:

(23) $$DHC_{\exp}=\frac{1}{b}\ln\frac{\Delta p_{f}}{\Delta p_{i}}$$

However, a limitation of this formula is that the filter dust loading behavior must fit the exponential curve. For those filters, which are better described by the polynomial function, an error can be introduced into this calculation—the same way it can be treated using polynomial fitting. However, obtaining an analytical solution of a 4th order polynomial is rather complicated, like the 3rd order polynomial. Based on the coefficient of determination and minimal differences of average pressure drop calculated using polynomials of different orders, we can simplify it to a polynomial of the 2nd order. Thus, the simplest way is to fit the pressure drop/dust load curve with a 2nd order polynomial, i.e., a quadratic function, so we can then solve a quadratic equation, the solution of which is simpler and can be expressed analytically. Based on the value of discriminant (*D*), we can obtain one real solution (*D* = 0), two different real solutions (*D* > 0), or no real solutions (*D* < 0, complex associated solutions). Equation (8) can be simplified from 4th to 2nd order polynomial:

(24) $$\Delta p\left(m\right)=ym^{2}+zm+\Delta p_{i}$$

Putting conditions (19)–(21) and rearranging, we get:

(25) $$yDHC^{2}+zDHC+\left(\Delta p_{i}-\Delta p_{f}\right)=0$$

This can be solved as a quadratic equation, taking only the non-negative roots to represent the *DHC_pol_* (DHC calculated from polynomial fit parameters), as follows:

(26) $$DHC_{\mathrm{pol}}=\frac{\sqrt{z^{2}-4y\left(\Delta p_{i}-\Delta p_{f}\right)}-z}{2y}$$

**Figure 6:**
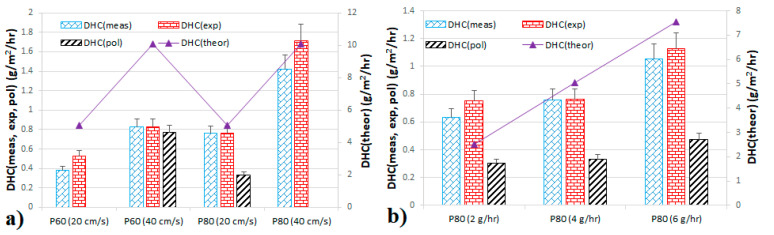
Dust-holding capacity per cake pressure drop of different membranes at different permeate velocities at a constant loading rate of 4 g/h (**a**) and P80 membrane at 20 cm/s at different loading rates (**b**), DHC measured (*DHC*_meas_), DHC from exponential fit (*DHC*_exp_) and DHC from polynomial fit (*DHC*_pol_) are at the major axis, DHC theoretical (*DHC*_theor_) is at the minor axis.

**Figure 7:**
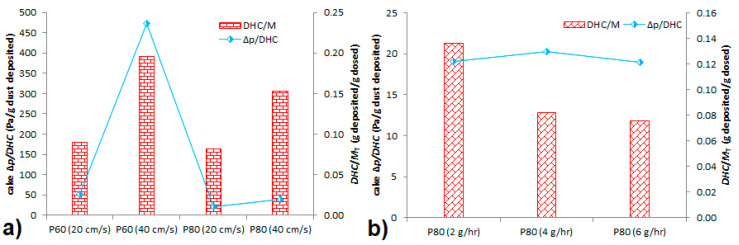
Cake pressure drop increase per gram of dust deposited and a ratio of DHC to total mass of dust dosed (*M*) for different membranes at different permeate velocities at a constant loading rate of 4 g/h (**a**) and P80 membrane at 20 cm/s at different loading rates (**b**).

Figure 6 shows a comparison of DHC obtained experimentally, theoretically calculated and obtained from different curve fitting in different experimental conditions. To better compare among different experimental conditions, DHC was related to unit membrane area and time. Generally, DHC increased with increasing flowrate (Figure 6a). This is in disagreement with results obtained for planar fibrous filters, where increasing the flowrate decreased filter DHC [27,28,29] or had practically no effect [30]. In the planar filter media, higher flowrate (face velocity) can cause weaker cake consolidation due to shorter time for depositing particles to arrange into a denser cake structure. The resulting dust cake is thus more porous and has lower weight. Here, the particles gradually deposit on the membrane surface and the cake is denser at the suction end of the membrane (i.e., where the membrane is connected to the fan) and then redistributed along the fiber length in the direction of increasing pressure drop. Thus, there is practically no cake at the dead end of the fiber [9]. However, at the suction end, the higher face velocity causes more particles to deposit, but it is not still enough to create a more porous dust cake. Therefore, the effect of flowrate is converse compared to planar filter media. This is confirmed by the *DHC/M*_T_ ratio (a ratio of deposited dust to total dust fed in the filtration chamber), which increased with increasing flowrate (Figure 7a). However, it is also important to note that the experiments were conducted in a narrow flowrate range so further research is necessary, also due to the pressure drop effects along the fiber length, which can be significant. This is confirmed in Figure 7a where the cake pressure drop per gram of deposited dust is different even though it should be practically the same as the same mass of particles should cause the same pressure drop increase. The pressure drop fluctuations are caused by the significant effect of inner fiber diameter, which is described by the Hagen–Poiseuille equation [31].

At different loading rates (dust concentrations), the dust-holding capacity increased (Figure 6b). This is contrary to that observed for depth filter media [32], in which DHC decreased with increased dust concentration or for composite filter, where the concentration had no effect on DHC [29]. As these experiments were conducted at the same flowrate but at different dust concentrations, we can see what was noted above that the cake pressure drop increase per gram of dust deposited is the same (Figure 7b), no matter what the dust concentration. It was 19.02, 20.24, and 19.00 Pa/g for dust loading rate of 2, 4, and 6 g/h, respectively. However, the decreasing *DHC/M*_T_ ratio (Figure 7b) with increasing dust concentration is not in accordance with the results presented in Figure 6b. According to Figure 6b, the *DHC/M*_T_ ratio (Figure 7b) should also increase. Thus, there is probably influence of the final dust load (which was due to experimental conditions different) or the increasing trend of DHC with dust load is only at an early stage of membrane dust fouling. This would mean that the trend in Figure 6b would be reversed if the experiments were further prolonged. However, this would be possible only in planar filter geometry. It is a problem with hollow fibers as the cake cannot be retained on the membrane surface and falls off. Hence, further work is necessary especially for filtration on a long-term basis and with concentrations common for general filtration applications. Here, we used extremely high dust concentrations to fasten the membrane fouling, which could also have an effect on DHC.

## 5. Conclusions

This work tried to analyze average pressure drop of hollow-fiber membranes during dust filtration and to outline dust-holding capacity of the same. Average pressure drop based on experimentally obtained values of initial and final pressure drop varied depending on the calculation model used. The same was true when using different fitting of dust loading curves. Hence, the selection of the right model to calculate the average pressure drop during the filter operation is of critical importance as this can cause over/underestimating of the filtration system design. Dust-holding capacity was outlined and the behavior was different compared to planar/pleated filter media. Increasing the flowrate, DHC increased, the same was true when increasing the dust concentration. It would be appropriate to conduct longer experiments to observe further characteristics. However, further prolongation of the experiments would probably not make sense as the structure of the cake deposited would not mechanically withstand further loading and would probably fall off of the membrane fibers’ surface. This is, however, another advantage of the hollow fiber membrane geometry, i.e., a self-cleaning ability. This work contributes to general knowledge about loading behavior and dirt holding capacity of membrane filters, which are increasingly being used in high efficiency filtration. Such applications include compressed air treatment, microelectronics, medical gases purification, and other related industries.

## Figures and Tables

**Table 1:** Basic parameters of the hollow-fiber membranes.

Hollow-Fiber Membrane	P60	P80
Fiber outer diameter (µm)	300	620
Number of fibers	1380	300
Active length (mm)	730	730
Surface area (m^2^)	0.95	0.43
Initial pressure drop (Pa m^−2^)	1143 ± 13	307 ± 2

**Table 2:** Overview of the raw experimental data obtained at different experimental configurations.

Experiment		*DHC*_meas_ (g)	Δ*p*_i_ (Pa)	Δ*p*_f_ (Pa)	*M_T_* (g)	*t*_f_ (h)
4 g/h	P60 (20 cm/s)	16.2	1204	2072	180	45
	P60 (40 cm/s)	4.7	3239	5581	24	6
	P80 (20 cm/s)	15.7	714	1453	192	48
	P80 (40 cm/s)	21.4	1870	3789	140	35
20 cm/s	P80 (2 g/h)	9.0	717	1115	66	33
	P80 (4 g/h)	15.7	714	1453	192	48
	P80 (6 g/h)	13.6	717	1318	180	30

## Data Availability

Not applicable.

## Nomenclature

*a*	a curve fitting parameter
*A*	membrane surface area
*Ar*	Archimedes number
*b*	a curve fitting parameter
*B*	a constant in relationship for pressure drop at filter exchange
*C*_up_	upstream particle concentration
*d*_p_	particle diameter
*D*	discriminant
*DHC*	dust-holding capacity
*DHC*_meas_	experimentally measured dust-holding capacity
*DHC*_exp_	dust-holding capacity from exponential fit
*DHC*_pol_	dust-holding capacity from polynomial fit
*DHC*_theor_	theoretical dust-holding capacity
*g*	gravitational acceleration
*H*	humidity
*K*	constant describing dust and/or filter type
*m*	dust accumulation in filter
*m*_HFM_	instantaneous mass of dust deposited on HFM
*m*_load_	dust loading rate
*m(t)*	mass of dust on filter at a given time
*M*	final dust loading
*M*_T_	total amount of dust dosed to the chamber
∆*p*(*m*)	pressure drop as a function of dust load
∆*p*(*t*)	pressure drop as a function of time
∆*p*_avg_	average pressure drop
∆*p*_c-o_	pressure drop at change out of the filter
∆*p*_f_	final pressure drop
∆*p*_i_	initial pressure drop
*Q*	air flowrate
*Re*	Reynolds number
*t*	instant time of filtration
*t*_f_	final time of filtration
*T*	temperature
*v*	curve fitting parameter
*x*	curve fitting parameter
*y*	curve fitting parameter
*z*	curve fitting parameter
*η*	filtration efficiency
*µ*	fluid viscosity
*ν*_p_	sedimentation velocity
*ξ*	friction coefficient
*ρ*	fluid density
*ρ*_p_	particle density
